# Critical Analysis of the Effect of Antiresorptive Drugs on Osteonecrosis Associated With Dental Implants: An Umbrella Review

**DOI:** 10.7759/cureus.71506

**Published:** 2024-10-15

**Authors:** Mansi Dahiya, Pankaj Dhawan, Sapna Rani, Vidushi Saxena

**Affiliations:** 1 Department of Prosthodontics, Manav Rachna Dental College, Faridabad, IND; 2 Department of Prosthodontics and Implantology, Manav Rachna Dental College, Faridabad, IND

**Keywords:** antiresorptive drugs, bisphosphonate, bisphosphonate-associated osteonecrosis, bronj, dental implant failure, mronj

## Abstract

The overview was carried out to evaluate the impact of antiresorptive drug (ARD) therapy in patients undergoing dental implant treatment who significantly experience medication-related osteonecrosis of the jaw (MRONJ) or not. A comprehensive electronic database search within the timeline of 2014-2024, restricted to the English language, was performed in database search engines like Google Scholar, Web of Science, PubMed, and Scopus. The articles were screened using the specific Medical Subject Headings (MeSH) terms and Boolean operators 'OR' and 'AND'. Studies with patients having a history of ARD before, during, or following implant placement were included in the systematic review, without or with meta-analysis. The AMSTAR 2 (A MeaSurement Tool to Assess systematic Reviews) tool was utilized to evaluate the quality of the papers. This review was registered in the International Prospective Register of Systematic Reviews (PROSPERO) database (CRD42024583017). There were 10 systematic reviews as per eligibility criteria. The total number of failed implants was 524, out of which the incidence of MRONJ was between the ranges of 2% and 17%. According to the AMSTAR 2 tool, 20% of the studies were rated as medium quality, 60% were rated as low quality, and 20% were rated as critically low quality. Three meta-analyses were observed for the event "MRONJ," and it found no statistically significant differences between dental implant users with ARD and non-ARD participants. The corroborative evidence presents that patients undergoing dental implants with ARD history significantly experience MRONJ if there is a lack of strategic planning of implant placement.

## Introduction and background

Dental implants have become a preferred treatment as it is the "best of both worlds" for missing teeth. The durability and significant preservation of bone and enhanced functional or aesthetic outcomes make it a more permanent solution when compared with other prosthetic options. However, in the era of fixed prosthodontics, there are few clinical scenarios wherein the indication and prognosis of dental implants should be weighed in contrast to their failure or complications [1]. As many studies suggest, immunocompromised patients with ongoing anti-cancer therapy, with severe bleeding disorders, or on intravenous (IV) bisphosphonate therapy pose a complete antipathy to dental implant therapy due to its highest rate of failure [2]. Bisphosphonate is the most commonly used antiresorptive drug (ARD). ARD therapy (e.g., alendronate, risedronate, ibandronate, pamidronate, and zoledronate) is the treatment modality advised for patients suffering from osteoporosis, bone metabolic diseases like Paget, or cancer [3]. It alters bone physiology by reducing osteoclastogenesis, increasing osteoclast apoptosis, decreasing bone resorption and osteoblastogenesis, and thus decreasing bone formation. All of this activity hinders the physiology of osseointegration [1]. The dental implant modality among those who have previously taken antiresorptive medications has attracted varied opinions. Kün-Darbois and Fauvel's study [4] states that bisphosphonate has no role in dental implant failure. In contrast, Li and Leung [5] inferred that osteonecrosis occurred after implant placement with a background of bisphosphonate intake. Medication-related osteonecrosis of the jaw (MRONJ) is defined in individuals with a history of antiresorptive or antiangiogenic medication treatment as exposed bone or bone that can be probed through an intraoral or extraoral fistula in the maxillofacial region that has lasted for more than eight weeks. The intake of ARDs is related to the potential failure of dental implants due to MRONJ [4]. Rebelo et al. [6] reported an MRONJ complication in six of 68 associated implants, giving a failure rate of 8.8%. The incidence of MRONJ depends upon various factors like the route of administration, the time frame between medication and placement of the dental implant, and risk and other comorbidities. de-Freitas et al. [7] reported the incidence of osteonecrosis in 19 patients with comorbidities like hypertension and diabetes mellitus. Rebelo et al. [6] suggested that MRONJ risk is higher in the presence of IV bisphosphonate than with the oral or subcutaneous route of administration. There is an uncertain consensus regarding MRONJ as a noteworthy consequence in patients with ARD history before, during, or following implant placement. Thus, with a comprehensive understanding and evaluation, this study aims to investigate the impact of ARD treatment in dental implant patients with a significant incidence of MRONJ.

## Review

Methods

Preferred Reporting Items for Systematic Reviews and Meta-Analyses (PRISMA) was followed in the development of this overview protocol, which has been registered in the International Prospective Register of Systematic Reviews (PROSPERO) database (CRD42024583017).

Focused Question

Does ARD treatment significantly manifest MRONJ in individuals receiving dental implants?

The PICOS (patient, problem, or population; intervention; comparison, control, or comparator; outcome(s); study type) for this umbrella review was as follows: for P, patients undergoing dental implant therapy for missing teeth; for I, patients with a history of ARD intake prior, during, or after dental implant placement; for C, patients without a history of ARD; for O, MRONJ; and for S, a systematic review with or without meta-analysis.

Search Strategy

A thorough approach to literature search was taken to find all pertinent systematic reviews and meta-analyses examining the substantial outcome of antiresorptive medications on MRONJ in patients receiving dental implants. An electronic search of databases such as Google Scholar, Scopus, Web of Science, and PubMed/MEDLINE was conducted. Boolean operators for the Medical Subject Headings (MeSH) phrases listed below were employed in the search method (Table 1). To determine eligibility, two investigators (MD and SR) independently revised titles and abstracts of systematic papers and meta-analyses that made the shortlist. The complete texts of the papers that the titles and abstracts deemed relevant were then evaluated. The same two researchers manually searched the references in all accessible systematic reviews and meta-analyses. Two authors assessed each of the chosen papers to determine whether or not they should be included in this comprehensive evaluation. Conflicts were settled by discussion with a third investigator (PD) and by consensus.

**Table 1:** Search strings utilized across the databases MeSH: Medical Subject Headings; MRONJ: medication-related osteonecrosis of the jaw

Database	Equation implemented	Filters
PubMed	(diphosphonates [MeSH Terms]) OR bisphosphonates [MeSH Terms]) OR clodronate [MeSH Terms]) OR etidronate [MeSH Terms]) OR alendronate [MeSH Terms]) OR Antiresorptive agents [MeSH Terms]) OR Dental implant associated Osteonecrosis [MeSH Terms]) OR ibandronate [MeSH Terms]) OR “bisphosphonate-associated osteonecrosis of the jaw” [MeSH Terms]) OR MRONJ OR Bisphosphonate related MRONJ) OR Dental implant related-MRONJ) OR zoledronate AND (osseointegration OR dental implants OR bisphosphonate related complications [MeSH Terms])	In English, from January 2014 to May 2024, humans
Scopus	ALL ((diphosphonates OR bisphosphonates OR clodronate alendronate OR risedronate OR bisphosphonate-associated AND osteonecrosis AND jaw) OR Antiresorptive agents OR dental implant associated MRONJ OR bisphosphonate AND dental implant OR Dental implant associated osteonecrosis OR ibandronate OR (minodronate OR zoledronate) AND (osseointegration OR dental AND implants OR MRONJ)	In English, from January 2014 to May 2024, humans
Web of Science	diphosphonates OR bisphosphonates OR clodronate OR etidronate OR alendronate OR pamidronate OR risedronate OR ibandronate OR (bisphosphonate-associated MRONJ AND osteonecrosis AND of AND the AND jaw) OR Dental implant OR associated OR osteonecrosis OR Bisphosphonate related MRONJ) AND (osseointegration OR dental implants) AND failure (All Fields)	In English, from January 2014 to May 2024, humans
Google Scholar	"Dental implant"[Mesh] OR “bisphosphonate” AND related AND ‘osteonecrosis” AND of AND the AND jaw, “bisphosphonate” AND “dental” AND implant, bisphosphonate OR dental OR implant, “antiresorptive” AND “drugs” AND dental AND implant, antiresorptive agent, antiresorptive AND drugs AND implant AND complication, antiresorptive OR drugs OR implant OR complication, bisphosphonate-associated AND “osteonecrosis” AND of AND the AND jaw, “antiresorptive” AND therapy AND in AND dental AND implant, “antiresorptive” OR therapy OR in OR dental OR implant, “osseointegration” AND “antiresorptive” AND agent, “osseointegration” OR “antiresorptive” OR agents	In English, from January 2014 to May 2024, humans

Eligibility Criteria

The patients with a history of ARD therapy before, during, or after dental implant placement for a missing tooth or teeth, systematic reviews with or without meta-analysis, studies involving patients receiving benign and malignant treatment, and reviews published within the time period of January 2014 to July 2024 were retrieved, and patients indicated for dental implant prosthesis for missing teeth were all involved in the following systematic review and meta-analysis. The following studies were excluded: papers published in a language other than English, studies failing to specify MRONJ as an outcome, in vitro or animal studies, primary or original clinical research, narrative review or scoping reviews, expert opinions, short communications, and editorials.

Data Extraction and Collection

After reading each abstract separately, the two authors (MD and SR) selected the most pertinent ones to receive the full-text screening. In the event of uncertainties or conflicts, a third reviewer (PD) was consulted. A thorough search was also conducted of the included review paper for any titles pertinent to this umbrella review. There were no limitations on the quantity or kind of studies that could be found in the included systematic reviews. The two reviewers (MD and SR) provided the majority of the review data; however, in cases of confusion, they consulted a third reviewer (PD), and in cases of further conflict, a fourth critic (VS) was consulted. In this umbrella study, systematic reviews yielded the following results: details such as author, publication year, references, and study quality are provided. The number and design of studies included the type of antiresorptive medication agent consumption and mode of drug intake, the number of unsuccessful implants in patients with antiresorptive medication and follow-up, and the main outcomes. The outcomes addressed in this review were MRONJ, bisphosphonate-related osteonecrosis of the jaw (BRONJ), implant survival or failure, implant loss, and MRONJ-related peri-implantitis.

Risk of Bias Assessment Protocol/Quality Assessment

The included systematic reviews and meta-analyses were assessed for methodological quality using the AMSTAR 2 (A MeaSurement Tool to Assess systematic Reviews) program. Three examiners (MD, SR, and PD) independently reviewed the included reviews. Examiners responded to the 16 questions of the AMSTAR 2 checklist for all systematic reviews by selecting "yes" or "partial yes" or "no" or "not relevant" as applicable. The information for the included publications was acquired from systematic papers and meta-analyses. After completing the AMSTAR 2 assessment, systematic reviews were rated as high, moderate, low, or critically low quality [6]. After completing this task individually, the three scrutineers (MD, SR, and PD) met to make a consensus decision for each research; any remaining disagreements were handled by discussion with a fourth investigator (VS).

Results 

Study Selection

**Figure 1:**
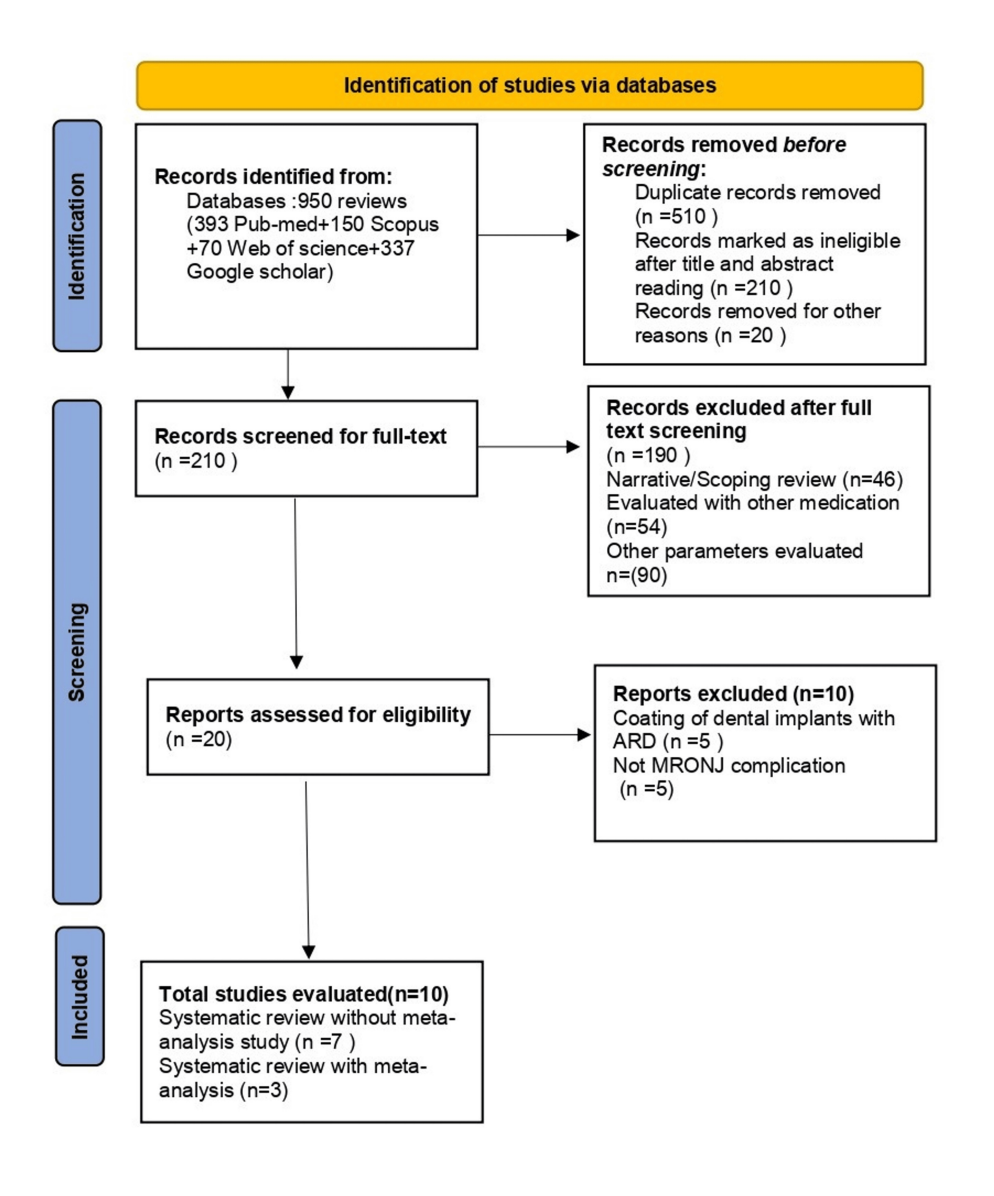
Flowchart of PRISMA selection of articles following the search strategy PRISMA: Preferred Reporting Items for Systematic Reviews and Meta-Analyses

Figure 1 represents the flowchart following PRISMA guidelines for systematic paper selection with or without meta-analysis. The search engine identified 950 eligible papers from all mentioned electronic databases. The two reviewers (MD and SR) independently searched eligible articles after screening the title and abstract. The duplicate articles from different databases and 10 ineligible articles due to the inability to meet the inclusion criteria were removed as presented in Table 2. Ten articles were retrieved after screening full-text papers for the comprehensive evaluation of this umbrella review.

**Table 2:** List of exclusion studies MRONJ: medication-related osteonecrosis of the jaw

Study	Reason for exclusion studies
Kellesarian et al. (2017) [8]	Coating of dental implant with antiresorptive drug
Fiorillo et al. (2022) [9]	With no MRONJ outcome
Martins et al. (2023) [10]	This review didn't include dental implant
Nisi et al. (2024) [11]	The outcome included peri-implantitis-induced MRONJ
Chappuis et al. (2018) [12]	Only seven studies included bisphosphonate intake with no MRONJ outcome
Limones et al. (2020) [13]	Review studies the comparison between the incidence of MRONJ when denosumab and zoledronic acid are used
Pishan et al. (2024) [14]	The review included only the oral route of drug administration
Aljohani et al. (2017) [15]	Patients with comorbidities like hypertension, diabetes mellitus, autoimmune disease, and smoking were included in the review
Goker et al. (2021) [16]	The inclusion criteria didn't include the population requiring dental implant treatment for missing teeth
He et al. (2020) [17]	Narrative review

Characteristics of Included Reviews

Table 3 and Table 4 summarize the relevant findings from the systematic papers (n=7) and meta-analyses (n=3) of the 10 included studies. We inspected four electronic databases (Google Scholar, Web of Science, PubMed, and Scopus) from January 2014 to March 2024. Case series, cohort studies, and case-control studies were among the searches conducted, along with controlled/randomized clinical trials and surveys, retrospective and prospective. The previous systematic review analyzed a total of 171 studies. The range of dental implants inserted in ARD patients was 38.5-3583 (mean=1830). The most common ARD agent intake was alendronate, risedronate, ibandronate, pamidronate, denosumab, clodronate, and bisphosphonates. The administration options included oral, IV, and subcutaneous (SC). Six systematic reviews evaluated the influence of ARD on dental implants if taken prior [8,18-22]. One systematic review assessed if ARD was taken after dental implant placement on the occurrence of MRONJ [23] and none during the implant placement phase. The total number of failed implants ranged between one and 58 implants, whereas failed implants due to MRONJ ranged between 0.4% and 17%. The time duration between the implant placements ranged between 24 and 111.5 months (mean=511.1 months). All the studies were retrieved from only human study data. Meta-analysis was performed in only three of 10 systematic reviews. The only reviews [12,20,23] that conducted a meta-analysis on "implant failure or peri-implant marginal bone loss" events demonstrated a statistically insignificant result (Table 5). Due to the heterogeneity of data, as detected by the I² test and odds ratio (OR), the main finding of these meta-analyses didn't help in the interpretations.

**Table 3:** Study characteristics and main finding of systematic reviews meeting the eligibility criteria ARD: antiresorptive drug; MRONJ: medication-related osteonecrosis of the jaw; BP: bisphosphonate; BRONJ: bisphosphonate-related osteonecrosis of the jaw; IV: intravenous

Study/year	No. of included studies (n)	Number of implants in ARD users	Design of studies included	Antiresorptive agents	No. of failed implants	Outcome	Follow-up period	Main finding
Sher et al. (2021) [19]	29	14-1267	Cohort studies, case-control studies, case series, retrospective, cross-sectional studies, prospective	Alendronate, risedronate, ibandronate, combination of alendronate and ibandronate	22	MRONJ, implant loss	0.3-12.2 years	Patients with a history of BP treatment are at risk of developing MRONJ after implant placement. Patients treated with denosumab for osteoporosis have a negligible risk of developing MRONJ after implant placement
Stavropoulos et al. (2018) [20]	36	8-61	Case series, cohort studies, case-control studies, controlled/randomized controlled clinical trials, retrospective and prospective studies	Alendronate, risedronate, ibandronate, pamidronate, denosumab, clodronate	1	MRONJ, implant loss, marginal bone loss	0.3-16 years	MRONJ in patients on BP for osteoporosis appeared in 70% of the cases >36 months after the start of drug intake, while in patients with cancer, MRONJ appeared in 64% of the cases ≤36 months after the first BP intake
Rebelo et al. (2023) [6]	13	163	Retrospective study, case reports and series, and prospective study	Orally alendronate, risedronate, and ibandronate, administered intravenously zoledronate, clodronate, and pamidronate	81	MRONJ, implant failure	12-48 months	A high mean failure rate of implant osseointegration (49.96%) was found, regardless of the generation of BPs used. Moreover, the failure rate was lower in patients using second-generation BP (alendronate and pamidronate) and was higher with the IV administration compared to the oral administration of BP
Li and Leung (2024) [5]	8	445	Case series, retrospective cohort studies	Alendronate, risedronate, zoledronate	92	MRONJ, peri-implantitis	Six months postoperatively	It is imperative that patients prescribed with ARDs are equipped with comprehensive information regarding the risks associated with MRONJ, particularly its localized impact around osseointegrated implant sites. A longer-term follow-up is recommended to identify and manage MRONJ around dental implants in an early manner
Gelazius et al. (2018) [21]	9	491	Randomized clinical trial assessments, case reports	Alendronate, risedronate, ibandronate, zoledronic acid, pamidronate	11	BRONJ	11.4-51.6 months	Patients treated IV could have a higher chance of developing implant-related BRONJ. Intraoral BP can be considered safe, if good pre- and postoperative care of the patient is in place
Anitua et al. (2024) [18]	14	52	Case reports, retrospective study, prospective study	Pamidronate, zoledronate, alendronate, ibandronate, or denosumab	57	MRONJ, peri-implant marginal bone stability, MRONJ recurrence	1-7 years	It could not be conclusively determined whether a fixed or a removable prosthetic solution is superior. Literature evidence suggests that wearing ill-fitting dentures is a risk factor for developing MRONJ. Therefore, in cases where a removable solution has been preferred, a strict clinical follow-up is highly advisable to readjust the prostheses when needed, to avoid soft tissue injuries
Papadakis et al. (2021) [22]	32	3583	Case series, retrospective study, prospective study	Alendronate, ibandronate, risedronate, clodronate, zoledronic acid, pamidronate	141	MRONJ	1-132 months	Perioperative antibiotic prophylaxis and careful treatment planning with frequent follow-ups and good oral hygiene are recommended. IV route ARD is an absolute contraindication. For patients with benign diseases or antibiotic prophylaxis or even in some cases, a drug holiday could be the strategy of choice
de-Freitas et al. (2016) [7]	15	1330	Case series, retrospective study, prospective study	Alendronate, risedronate, zoledronate, ibandronate, and pamidronate	113	BRONJ	1-132 months	The risk of developing BRONJ as well as occurring failure or loss of implant exists, and it is greater in patients under IV BP therapy. A complete medical history of the patient must be analyzed, and in case therapy with bisphosphonate is confirmed, the duration of treatment as well as the route of administration should be taken into consideration
Ata-Ali et al. (2016) [23]	8	1090	Case series, retrospective study, prospective study	Alendronate, risedronate, ibandronate, etidronate, tiludronate, zoledronic acid	6	MRONJ	4-89 months	Our results show that dental implant placement in patients receiving BP does not reduce the dental implant success rate. On the other hand, such patients are not without complications, and risk evaluation therefore must be established on an individualized basis, as one of the most serious though infrequent complications of BP therapy is BRONJ
Chappuis et al. (2018) [12]	7	400	Randomized controlled trials	Alendronate, risedronate, ibandronate, zoledronic	14	BRONJ	12-86.4 months	The use of oral BPs for the treatment of osteoporosis did not yield significance when analyzing their impact on implant failure. This finding is of special interest as oral BP intake was reported to be associated with a significantly higher risk of developing osteonecrosis of the jaw due to the blocking of osteoclastic activity

**Table 4:** Summary of main outcomes from the included systematic reviews ARD: antiresorptive drug; MRONJ: medication-related osteonecrosis of the jaw; BP: bisphosphonate; BRONJ: bisphosphonate-related osteonecrosis of the jaw; IV: intravenous

Author/year	No. of cases included	Indication of ARD	Route of administration	Type of ARD	ARD intake before/duration/after implant placement	No. of implant failure due to MRONJ	Time duration between implant placement and MRONJ	Site of MRONJ
Sher et al. (2021) [19]	6-235	Osteoporosis, hormone replacement therapy, malignancies	Oral, IV	Alendronate, risedronate, ibandronate, combination of alendronate and ibandronate	0.25-20.3 years prior	22	0-180 months	Posterior maxillary and mandibular arch
Stavropoulos et al. (2018) [20]	7	Osteoporosis, cancer, hormonal replacement therapy	IV, SC, oral	Alendronate, risedronate, ibandronate, pamidronate, denosumab, clodronate	1-12 months prior	1	0-223 months	Posterior maxilla and mandible
Rebelo et al. (2023) [6]	67	Osteoporosis, multiple myeloma, breast cancer, lung cancer, prostate cancer, knee cancer, and osteogenesis imperfecta	Oral and IV	Orally alendronate, risedronate, and ibandronate, administered intravenously zoledronate, clodronate, and pamidronate	Varied between patients who did not discontinue and patients who discontinued for two, three, or six months before surgery, with respective resumption for one, three, or eight months after surgery	7 implants out of 13	NA	NA
Li and Leung (2024) [5]	135	Osteoporosis, cancer-related condition	Oral, IV, SC	Alendronate, risedronate, zoledronate	NA	76	Mean time between ARD and initiation of MRONJ: 3-192 months	Posterior mandible, maxilla
Gelazius et al. (2018) [21]	25	Osteoporosis breast and prostate cancer, multiple myeloma, Paget disease	Oral, IV	Alendronate, risedronate, ibandronate, zoledronic acid, pamidronate	6-84 months prior	2	NA	Posterior mandible, maxilla, left and right lower canine
Anitua et al. (2024) [18]	25	Osteoporosis, cancer-related condition	NA	Pamidronate, zoledronate, alendronate, ibandronate, or denosumab	Prior to implant surgery	2	8-31 months	Mandible
Papadakis et al. (2021) [22]	1684	Osteoporosis, metastatic bone disease	Oral, IV	Alendronate, ibandronate, risedronate, clodronate, zoledronic acid, pamidronate	6-132 months prior to implant surgery	210	0-156 months	Posterior areas of the mandible and maxillary arches
de-Freitas et al. (2016) [7]	528	Osteoporosis, osteoarthritis and polymyalgia rheumatica, cancer-related patients	Oral, IV, or both	Alendronate, risedronate, zoledronate, ibandronate, and pamidronate	N/A	113	3-192 months	Posterior maxillary and mandibular arch
Ata-Ali et al. (2016) [23]	386	Osteoporosis osteopenia, cancer, and bone metastases	Oral, IV	Alendronate, risedronate, ibandronate, etidronate, tiludronate/oral, zoledronic acid	Duration of drug 16-72 months after implant placement	6	37-68 months after implant placement	Posterior mandible
Chappuis et al. (2018) [12]	400	Osteoporosis, osteoarthritis	Oral	Alendronate, risedronate, ibandronate, zoledronic acid	36 months prior	18	NA	NA

**Table 5:** Results of the meta-analysis for the events "implant failure" and "implant loss," reported by the three reviews that performed quantitative analysis Heterogeneity reported in individual reviews using the I² test and odds ratio (OR) included in the table

Study	Event	P-value	Risk ratio	95% CI	Heterogeneity
Stavropoulos et al. (2018) [20]	Implant loss	<0.01	2.7	0.00-0.007	I^2^=36.9%
Ata-Ali et al.(2016) [23]	Implant failure rate	0.156	2.18	0.01-51.21	OR=1.43
Chappuis et al. (2018) [12]	Implant failure rate	<0.01	2.02	2.67-6.39	I^2^=96.87%

Methodological Quality of Assessment

The assessment of the methodological quality of the included systematic reviews was done using the AMSTAR 2 tool [24,25]. Table 6 shows the AMSTAR 2 tool evaluating the quality grade of the systematic review included. The score varied from 1 to 10 points, with an average of 6 out of possible 16 points. Items 1, 2, 7, 8, and 9 scored positive for the included studies. According to the AMSTAR 2 tool, moderate quality was 20% out of all the studies; 60% low quality; and 20% critically low-quality evidence. However, no study recorded high-quality scores. Only three research papers [21,22,7] explicitly stated that the assessment process was established before the review (item 2). Five studies [6,5,21,18,12] used a standard technique to assess the risk of bias (item 9). Table 7 represents the result of the positive outcome of MRONJ in the included studies and the percent of the incidence of MRONJ out of failed implants.

**Table 6:** Assessment of the methodological quality of the systematic reviews included using the AMSTAR 2 instrument AMSTAR 2: A MeaSurement Tool to Assess systematic Reviews; PICO: population, intervention, comparison, and outcome; RoB: risk of bias; Y: yes; N: no; P/Y: partial yes; -: without meta-analysis

AMSTAR 2 tool items	Systematic review									
	Sher et al. (2021) [19]	Stavropoulos et al. (2018) [20]	Rebelo et al. (2023) [6]	Li and Leung (2024) [5]	Gelazius et al. (2018) [21]	Anitua et al. (2024) [18]	Papadakis et al. (2021) [22]	de-Freitas et al. (2016) [7]	Ata-Ali et al. (2016) [23]	Chappuis et al. (2018) [12]
1. Did the research questions and inclusion criteria for the review include the components of PICO?	Y	Y	Y	Y	Y	Y	Y	Y	Y	Y
2. Did the report of the review contain an explicit statement that the review methods were established prior to conducting the review and did the report justify any significant deviations from the protocol?	P/Y	P/Y	P/Y	P/Y	Y	P/Y	Y	Y	P/Y	P/Y
3. Did the review authors explain their selection of the study designs for inclusion in the review?	N	N	N	N	N	N	Y	Y	Y	Y
4. Did the review authors use a comprehensive literature search strategy?	N	P/Y	P/Y	P/Y	P/Y	P/Y	P/Y	Y	Y	Y
5. Did the review authors perform study selection in duplicate?	Y	N	N	N	N	Y	N	Y	Y	Y
6. Did the review authors perform data extraction in duplicate?	Y	N	N	N	N	Y	N	N	Y	Y
7. Did the review authors provide a list of excluded studies and justify the exclusions?	Y	Y	Y	P/Y	Y	P/Y	Y	Y	Y	Y
8. Did the review authors describe the included studies in adequate detail?	Y	Y	Y	P/Y	P/Y	P/Y	P/Y	P/Y	P/Y	Y
9. Did the review authors use a satisfactory technique for assessing the RoB in individual studies that were included in the review?	P/Y	P/Y	Y	Y	P/Y	Y	Y	P/Y	P/Y	Y
10. Did the authors report on the sources of funding for the studies included in the review?	N	N	Y	N	N	N	N	P/Y	P/Y	N
11. If meta-analysis was performed, did the review authors use appropriate methods for the statistical combination of results?	-	N	N	-	-	-	-	N	N	Y
12. If meta-analysis was performed, did the review authors assess the potential impact of RoB in individual studies on the results of the meta-analysis or other evidence synthesis?	-	N	N	-	-	-	-	-	Y	Y
13. Did the review authors account for RoB in individual studies when interpreting/discussing the results of the review?	-	N	Y	-	-	-	-	-	N	N
14. Did the review authors provide a satisfactory explanation for, and discussion of, any heterogeneity observed in the results of the review?	-	N	N	-	-	-	-	-	Y	Y
15. If they performed quantitative synthesis, did the review authors carry out an adequate investigation of publication bias (small study bias) and discuss its likely impact on the results of the review?	-	N	N	-	-	-	-	-	N	Y
16. Did the review authors report any potential sources of conflict of interest, including any funding they received for conducting the review?	-	N	Y	-	-	-	-	-	Y	N
Total score	6	4.5	8	4	4.5	6	6	7.5	11	11.5
Quality of evidence	Low	Critically low	Low	Critically low	Low	Low	Low	Low	Moderate	Moderate

**Table 7:** Effect of ARDs in relation to the outcome MRONJ ARDs: antiresorptive drugs; MRONJ: medication-related osteonecrosis of the jaw; +: present; -: absent

S. no.	Author	MRONJ	Percent of MRONJ out of failed implants (%)
1	Sher et al. (2021) [19]	+	3
2	Stavropoulos et al. (2018) [20]	+	2
3	Rebelo et al. (2023) [6]	+	4
4	Li and Leung (2024) [5]	+	17
5	Papadakis et al. (2021) [22]	+	0.4
6	Anitua et al. (2024) [18]	+	4
7	Gelazius et al. (2018) [21]	+	4
8	de-Freitas et al. (2016) [7]	+	8
9	Ata-Ali et al. (2016) [23]	+	0.5
10	Chappuis et al. (2018) [12]	+	4

Discussion

The current overview focus question highlights the considerable MRONJ occurrence in ARD users undergoing dental implant therapy. Although there is an existing systematic literature regarding ARD's impact on dental implant survival, there is a lack of consensus about implant failure remarkably due to MRONJ. By the amalgamation of systematic reviews, the present study evaluated and assessed the most evident inference from the data. This umbrella review study aimed to analyze and combine data on the relationship between ARD and MRONJ in dental implant patients. Among the included systematic reviews, the primary studies analyzed were quite distinct, ranging from numbers 7 to 36. A thorough elaborative search strategy was used to increase the reliability of evident results and reduce the risk of bias. The history of ongoing ARD therapy in an individual reflects inhibited osteoclast function and disrupted bone turnover processes, which remarkably led to the hypothesis that it would hinder the normal osseointegration process. Furthermore, the surgical intervention of dental implants along with the inhibited bone remodeling in such patients results in MRONJ. Keeping this in mind, the road map of the review included the design and number of studies, the type of ARDs, the indications of ARDs, the drug administration route, the period of drug intake, the number of failed implants due to MRONJ, the site of MRONJ, and the follow-up period (Table 3, Table 4). AMSTAR 2 is a proposed device for assessing the quality grade of review papers [24]. This tool is widely used for examining systematic reviews and meta-analyses of research, whether randomized or not. The tool includes seven primary domains (items 2, 4, 7, 9, 11, 13, and 15) that critically assess the quality of the paper and help to draw valid and reliable conclusions. Our umbrella review according to AMSTAR 2 reported two reviews [23,12] as moderate quality, six reviews [6,17-19,21,22] as low quality, and two reviews [20,5] as critically low quality (Table 5).

The commonly used ARDs were alendronate, risedronate, ibandronate, pamidronate, denosumab, clodronate, and bisphosphonates. In the included studies, ARDs were indicated for benign diseases like osteoporosis, osteoarthritis, bone metabolic disorders, and malignant diseases like breast, prostate, multiple myeloma, and metastatic bone disease.

The result of the overview showed that cancer patients were more prone to MRONJ than patients who had benign diseases like osteoporosis, Paget disease, or hormonal replacement therapy. In a systematic review by Rebelo et al. [6], the use of oral or IV ARDs for osteoporosis before implant placement showed seven implant losses out of 13 due to MRONJ. Similar results were seen by Li and Leung [5] in their study which inferred ARD for osteoporosis where four implants failed out of 12 due to MRONJ. Unlike in malignant diseases, the incidence of MRONJ was greater with the IV drug route. According to de-Freitas et al. [7], the use of ARD to treat cancer with the IV drug route showed 100% implant loss due to MRONJ. Moreover, the route of ARD's administration happens to pose a greater likelihood of MRONJ; the intravenous route was reported to be associated with a maximum number of MRONJ cases. In Gelazius et al. [21], the use of IV bisphosphonate for 72 months showed 100% failure of all dental implants due to MRONJ, whereas in Papadakis et al. [22], the study presents 100% survival of dental implants when oral bisphosphonate was given for 20.5 months.

Another inference drawn was that the duration between implant placement and initiation of MRONJ was 3-19 months with an average of 99.5 months. Ata-Ali et al. [23] demonstrated that this is due to disrupted bone modeling and trabecular microarchitecture, inhibition of angiogenesis, and high susceptibility to infection resulting in osteonecrosis. Sher et al. [19] results show a similar conclusion that in 830 patients, 2841 implants were placed, out of which MRONJ cases were found to be 102 with a prevalence of 12% in 0-180 months. It was stated that it was mainly due to peri-implant infection and inflammation.

The majority of dental implant failure out of all the systematic reviews was due to IV bisphosphonate in cancer-related patients with a duration of more than 50 months of therapy, and the most common site was reported to be the posterior mandible, followed by the posterior maxilla [4,6,7,18-23,26]. The incidence of MRONJ was minimal when the oral route of the drug was chosen, and drug holiday was also reported to be a good option to reduce the risk of MRONJ. According to a systematic review by Anitua et al. [18], oral ARDs had the least number of MRONJ cases. This study also recommended the use of the drug holiday concept for the patient on ARD and exposed for an extended four years. In this strategy, the ARD is discontinued to let the bone marrow recover till new osteoclasts are formed, so that they can attain former numbers before their decline. It was also observed that the time of ARD intake before, during, and after also influenced the incidence of MRONJ. Six systematic reviews [8,12,19-22] stated that cessation of ARD intake at least 12-132 months prior notably has a lower onset of MRONJ (4-14%). Two reviews [6,23] presented ARD intake after 8-72 months of implant placement with 100% implant success. The cumulative assessment of 10 systematic reviews showed that the total number of failed implants was 524, out of which the incidence of MRONJ was between the range of 2% and 17% (Table 6).

To reduce the prevalence of MRONJ, patients should be informed about the potential triggers of MRONJ. As Anitua et al.'s [18] study stated, ill-fitting dentures acted as a trigger for MRONJ in patients with dental implants. Comorbidities such as hypertension, ischemic heart disease, anemia, dementia, renal failure, diabetes, and smoking significantly increase the risk of developing MRONJ. The most commonly cited trigger factors were chemotherapy, corticosteroid use, and smoking [18]. According to Ciobanu et al. [25], MRONJ risk factors include chemotherapy, zoledronic acid intake, hypertension, and treatment length. Cancer survivors who underwent chemotherapy or had hypertension were more likely to acquire MRONJ. Another inference drawn out of the cumulative assessment to limit the occurrence of MRONJ is reinforcing and counseling patients with an urgent need to maintain proper oral hygiene and long monitoring. Chlorhexidine is recommended for lowering biofilm formation and inflammation during the early healing stages. Patients should also be told about the link between poor hygiene causing peri-implantitis and the initiation of MRONJ [25]. Moreover, prophylactic measures like antibiotic prophylaxis could be advocated to reduce the occurrence of MRONJ after surgical intervention. In 2014, Ata-Ali et al. [23] conducted a meta-analysis of 110 and stated that the use of antibiotics lowers implant failure by 66.9%. A likewise approach can be implemented by ARD's users to reduce the incidence of MRONJ. 

AMSTAR 2 assessed two reviews [12,23] as moderate quality, six reviews [6,17-19,21,22] as poor quality, and two reviews [20,5] as critically low quality (Table 6). This indicates that the review did not provide adequate details of the methodology, such as study protocol registration, appropriate literature search, justifying individual excluded studies, risk bias assessment of primary studies, and adequate meta-analytical methods.

Strengths and Limitations

The study's strengths include an unconstrained search method, eliminating duplicates, and extracting high-quality data. This is the first umbrella review on the relationship between the considerable incidence of MRONJ in ARD users undergoing dental implants. However, there are a few limitations to the overview; the retrieved papers included more cohort studies (retrospective and prospective), case reports, and case series, increasing the likelihood of bias. Secondly, many reviews had heterogeneity of data presentation; few mentioned the type and route of ARDs. Also, no time window was mentioned between implant insertion and the beginning of MRONJ [25], and few studies didn't mention the site of MRONJ [6]. Thirdly, based on the AMSTAR 2 score, only two reviews were of moderate quality. This probably signifies the inappropriate details of methodology and assessment of risk bias.

Clinical Implications

The clinical evidence shows that the chances of implant failure are high in ARD users, mainly due to MRONJ. There are contributing factors like dose, route, the time frame between drug intake and implant placement, and comorbidities like diabetes, cancer patients, or corticosteroid therapy that significantly increase the incidence of MRONJ. Since there is no method to detect the incidence of MRONJ, the individual patient should be clinically analyzed for the risk, and a diligent schematic approach should be followed. Patient awareness and knowledge regarding trigger factors, along with frequent follow-up, can incredibly reduce the onset of MRONJ.

Future Directions

Based on the data, it is suggested that a greater number of controlled trials and individual assessment of parameters should be encouraged in the future. The variability in study quality, as indicated by the AMSTAR 2 assessments, suggests that the conclusions drawn from some of these reviews should be viewed cautiously. Furthermore, research, particularly high-quality randomized controlled trials and long-term clinical studies with extensive follow-up, can help to corroborate the findings of these systematic reviews and prescribe regimens in ARD users with the lowest MRONJ incidence.

## Conclusions

This umbrella review reflects that there is a significant onset of MRONJ after ARD's use, notably in patients with IV route bisphosphonate for more than 45-60 months of duration. Moreover, comorbidities like smoking, diabetes mellitus, hypertension, anti-cancer therapy, and corticosteroid therapy make patients more prone to implant failure, mainly due to MRONJ. Since only clinical analysis can help to decide the prognosis of dental implants in ARD users, it becomes crucial to comprehensively plan implant placement and reinforce patients' awareness regarding oral hygiene and the need for frequent monitoring. It was also evident that antibiotic prophylaxis and drug-judicious use can help in improving dental implant survival and success outcomes.
